# LMP1 and Dynamic Progressive Telomere Dysfunction: A Major Culprit in EBV-Associated Hodgkin’s Lymphoma

**DOI:** 10.3390/v9070164

**Published:** 2017-06-27

**Authors:** Hans Knecht, Sabine Mai

**Affiliations:** 1 Division of Haematology, Department of Medicine, Jewish General Hospital, McGill University, Montréal, QC H3T 1E2, Canada; 2 Manitoba Institute of Cell Biology, The Genomic Centre for Cancer Research and Diagnosis, University of Manitoba, Winnipeg, MB R3E 0V9, Canada; smai@cc.umanitoba.ca

**Keywords:** LMP1, EBV, TRF2, telomere, shelterin, Reed–Sternberg cell, Hodgkin’s lymphoma, 3D TRF2/Telo-Q-FISH

## Abstract

Epstein–Barr virus (EBV)-encoded latent membrane protein 1 (LMP1) is expressed in germinal-center-derived, mononuclear Hodgkin (H) and multinuclear, diagnostic Reed–Sternberg (RS) cells in classical EBV-positive Hodgkin’s lymphoma (cHL). LMP1 expression in EBV-negative H-cell lines results in a significantly increased number of RS cells. In a conditional, germinal-center-derived B-cell in vitro system, LMP1 reversibly down-regulates the shelterin proteins, telomeric repeat binding factor (TRF)1, TRF2, and protection of telomeres (POT)1. This down-regulation is associated with progressive 3D shelterin disruption, resulting in telomere dysfunction, progression of complex chromosomal rearrangements, and multinuclearity. TRF2 appears to be the key player. Thus, we hypothesize that the 3D interaction of telomeres and TRF2 is disrupted in H cells, and directly associated with the formation of H and RS cells. Using quantitative 3D co-immuno-TRF2-telomere fluorescent in situ hybridization (3D TRF2/Telo-Q-FISH) applied to monolayers of primary H and RS cells, we demonstrate TRF2-telomere dysfunction in EBV-positive cHL. However, in EBV-negative cHL a second molecular mechanism characterized by massive up-regulation of TRF2, but attrition of telomere signals, is also identified. These facts point towards a shelterin-related pathogenesis of cHL, where two molecularly disparate mechanisms converge at the level of 3D Telomere–TRF2 interactions, leading to the formation of RS cells.

## 1. Introduction

LMP1 (latent membrane protein 1) is an Epstein–Barr virus (EBV) encoded multifunctional oncoprotein, discovered more than 30 years ago [1,2]. The role of EBV in neoplastic transformation, in particular the role of LMP1, has been known for a long time [3], and the multiple facets of LMP1–host cell interactions are ever increasing [4]. New directions and alternative scenarios in EBV research have very recently been addressed in detail [5]. However, a probably seminal discovery, the direct interaction of the telomere repeat binding factors TRF1 and TRF2 with EBV through three telomere repeat-like nonamer binding sites (TTAGGGTTA) within the episome maintenance element OriP [6,7], escapes the attention of traditional EBV reviews. This and other types of viral–host interaction, targeting the shelterin complex and thus telomeres, are a promising field of ongoing research [8,9]. Telomeric DNA and the shelterin complex consist of multiple (TTAGGG)n repeats ending in a single stranded-overhang of the G-rich 3′ strand, and a number of specific proteins, called shelterins, either bind telomeric DNA directly or are associated with telomeric chromatin, and are found on telomeres [10,11,12]. The six human shelterin proteins are TRF1 (telomeric repeat binding factor 1), TRF2 (telomeric repeat binding factor 2), POT1 (protection of telomeres 1), TIN2 (TRF1 interacting nuclear protein 2), TPP1 (TIN2 interacting protein 1), and RAP1 (repressor activator protein 1). TRF1, TRF2, and POT1 directly interact with telomeric DNA, whereas TIN2, TPP1, and RAP1 interact sterically with the former three to form the 3D telomeric complex (reviewed in [13]).

EBV is associated with classical Hodgkin’s lymphoma (cHL) in about 48% of cases, and the incidence of EBV-positive, LMP1-expressing cHL is significantly higher in Africa and South America compared to other regions [14]. EBV-positive cHL shows a higher incidence in children, in males, and in advanced clinical stages [14]. A negative impact on overall survival has been reported in children and young adults with advanced stage disease [15], and elderly women [16]. 

The molecular pathogenesis of cHL has markedly advanced through studies of EBV-negative Hodgkin cell lines derived from advanced stage disease patients. In EBV-negative cHL, constitutive activation of the nuclear factor kappa light-chain enhancer of activated B-cells (NF-κB) pathway, mutations in the genes coding for NF-κB inhibitors, and aberrant Notch signaling activity, play an important role in the formation of mononuclear Hodgkin (H) and diagnostic, multinuclear Reed–Sternberg (RS) cells (reviewed in [17]). The JAK/STAT signaling pathway is activated in H and RS cells either through cytokines, JAK2 chromosomal amplification/rearrangements, or inactivating mutations of SOCS1, a main inhibitor of STAT activity (reviewed in [18]). On the contrary, EBV-transformed lymphoblastoid cell lines (LCLs), also characterized by the capacity of infinite growth, have a diploid non-malignant genotype, are mononuclear, show an activated B-cell phenotype, and have a high spontaneous apoptosis rate [19,20]. The continuous proliferation of LCLs is possible through concert interaction of six Epstein-Barr nuclear antigens (EBNAs), three LMPs, and multiple microRNAs [21]. Obviously, since in EBV-associated cHL the only oncogenic protein expressed is LMP1, EBV-transformed LCLs are not suitable as models for EBV-positive cHL. An experimental, pathogenetic system for EBV-associated cHL would need an EBV-negative B-cell line expressing LMP1. 

Here, we focus on a recently developed experimental system for EBV-associated cHL and the dynamic three-dimensional (3D) LMP1–TRF2 interaction identified in the mononuclear Hodgkin (H) and diagnostic, multinuclear Reed–Sternberg (RS) cells—the malignant tumor cells of classical cHL [22]. 

## 2. Latent Membrane Protein 1 (LMP1) Induces Multinuclearity

In infectious mononucleosis (IM), lymph node biopsies performed for suspected malignancy reveal, amongst numerous EBER+ small mononuclear lymphocytes, a few bi- or multinucleated CD30-positive cells, indistinguishable morphologically from RS cells [23]. These cells are CD15-negative but express the B-cell specific transcription factors BOB.1 and OCT.2, which are absent in RS cells [24]. When the LMP1 oncogene is transiently expressed in the EBV-negative H-cell lines L-428 and HD-MyZ, as well as in the human embryonic kidney cell line 293, most of the LMP1 transfectants become multinucleated already after 48 h of culturing, with an increase up to 7 days, and a few large RS cells still identifiable at day 14 [25,26]. It is noteworthy to mention that the number of RS cells in H-cell lines (EBV-negative) is generally <5%, the bulk of cells being mononucleated H cells, and that after transfection with plasmid pSV2-LMP1, the number of LMP1-expressing RS cells and 293 polycaria gradually increases over time up to 70%. This multinuclearity is clearly LMP1-specific for it is not observed with the empty plasmid or after transient transfection of EBNA1 or EBNA2. All EBNA1 and EBNA2 transfectants remain mononuclear [3,26]. From these experiments, it was concluded that LMP1 is a powerful inducer of RS cells in H-cell lines, and of multinuclearity in the 293-cell line. However, the mechanism behind this transition/transformation remains to be elucidated. It had to be associated with basic mechanisms of cellular biology, since the changes were observed not only in quite differentiated cells (germinal-center-derived B-cells), but also in a cell line derived from embryonic kidney. Thus, we hypothesize that multinuclearity is associated with impairment of telomere function because H-cell lines harbor multiple chromosomal abnormalities as ectopic subtelomeres, jumping translocations, Robertsonian translocations, and duplications in the short arms of acrocentric chromosomes [27], and because large or multiple pericentrin structures are identified in RS cells of the H-cell line L-428 [28].

## 3. Assessment of 3D Telomere Dynamics

Telomeres are nucleoprotein complexes at the ends of chromosomes. Telomere DNA consists of multiple (TTAGGG)n repeats ending in a single-stranded overhang of the G-rich 3′ strand, and a number of specific proteins called shelterin which either bind telomeric DNA directly or are associated with telomeric chromatin [10,11,12,13]. 3D nuclear telomere organization is cell-cycle dependent, and telomeres assemble into a disk in the late G2 phase. However, in cancer cells this organization is disturbed and telomere aggregates are formed [29]. Telomere aggregates are defined as clusters of telomeres that are found in close association and cannot be further resolved as separate entities at an optical resolution of 200 nm. We have developed quantitative software that enables us to measure the 3D nuclear organization of telomeres [30]. This allows us to determine the 3D nuclear localization and distribution of telomeres, their size, their numbers, and the presence of telomere aggregates for each cell. Aggregates are hallmarks of cancer cells [31] and can be induced experimentally by c-myc overexpression, resulting in end-to-end telomere fusion of chromosomes and the initiation of subsequent breakage–fusion-bridge (BFB) cycles, resulting in dynamic and ongoing genomic instability [32]. Three-dimensional super-resolution imaging (3D-SIM) [33] identifies these aggregates partially as clusters of (extremely) small telomeres [9], so-called “t-stumps” [34], a further marker of cancer cells. This form of genomic instability is also observed after ex vivo EBV infection of human B-lymphocytes, and is associated with dysfunctional telomeres due to partial displacement of TRF2 [35], gain or loss of telomere signals [36], and low levels of TRF1, TRF2, and POT1 [37]. 

## 4. In Vitro Model for EBV-Associated Classical Hodgkin’s Lymphoma

**Figure 1:**
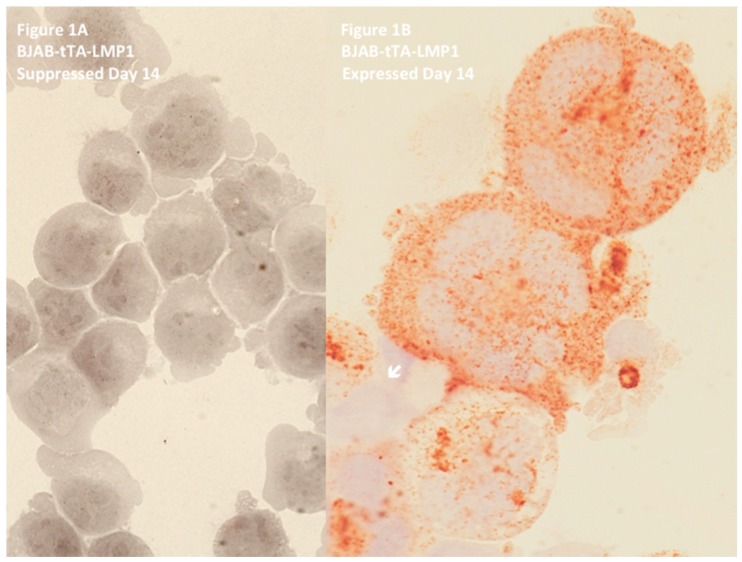
Latent membrane protein 1 (LMP1) expression in BJAB-tTA-LMP1 Burkitt’s lymphoma cells is associated with multinuclearity. Original magnification 640×, Zeiss AxioImager Z1 microscope (Zeiss, Toronto, ON, Canada). (**A**) LMP1-suppressed transfectants at day 14 still reveal uniform Burkitt cell morphology with only rare bi-nucleated or large mononuclear cells. Immunostaining with anti-LMP1 MoAb CS1-4 confirms successful LMP1 suppression through tetracycline. (**B**) LMP1-expressing transfectants at day 14 contain multiple Reed–Sternberg-like giant cells. Strong LMP1 expression is confirmed with anti-LMP1 MoAb CS1-4. Only one small mononuclear cell (arrow) appears not to express LMP1. Note several LMP1-positive vesicles (exosomes) at the surface of the top two polycaria. In vivo, such vesicles may influence the tumour microenvironment [48]. Photomicrograph performed in parallel during the experiments shown in Figure 2 of Lajoie et al. [46].

In EBV-positive cHL, the H and RS cells permanently express the LMP1 oncoprotein [38], or its 30 or 69 base-pair deletion variants [39]. Hodgkin’s lymphoma (EBV-positive or EBV-negative) is a germinal center-derived B-cell disease [22,40], and thus we hypothesized that every experimental cell culture system had to fulfill three criteria in order to mimic the conditions of EBV-associated cHL: (i) EBV-negative in order to avoid a latency III expression pattern, (ii) germinal center-derived B-cell origin, and (iii) inducibility of permanent LMP1 expression without leakage. These three conditions were unified in the EBV-negative, Diffuse Large B-Cell Lymphoma (DLBCL) cell line BJAB-tTA-LMP1 [41], a kind gift of Martin Rowe. BJAB does not have any proven rearrangement involving c-myc [42] and expresses only a low steady-state level of c-myc RNA [43]. In agreement with recent RNA expression profiling, BJAB is now accepted as a germinal center B-cell-derived (GCB)-DLBCL cell line [44,45]. In this tet-Off system, the stably transfected LMP1 oncogene is completely suppressed in the presence of tetracycline, but permanently expressed in the absence of the antibiotic. In the LMP1-suppressed culture, bi- and multinucleated cells comprise a maximum of 13% of the cells, and this percentage remains unchanged from day one until day 21 [46]. However, as shown in Figure 1, upon LMP1 expression, formation of RS-like multinucleated cells increases already after 48 h, impressively progresses up to over 30% at day 14, and remains stable until day 21 [46]. This difference is highly significant (*p* < 0.0001). Most LMP1+ RS-like cells contain three or more nuclei and are characterized by a high number of very short (<5000 arbitrary fluorescent units) and short telomeres (5000–15,000 arbitrary fluorescent units) [47].

**Figure 2:**
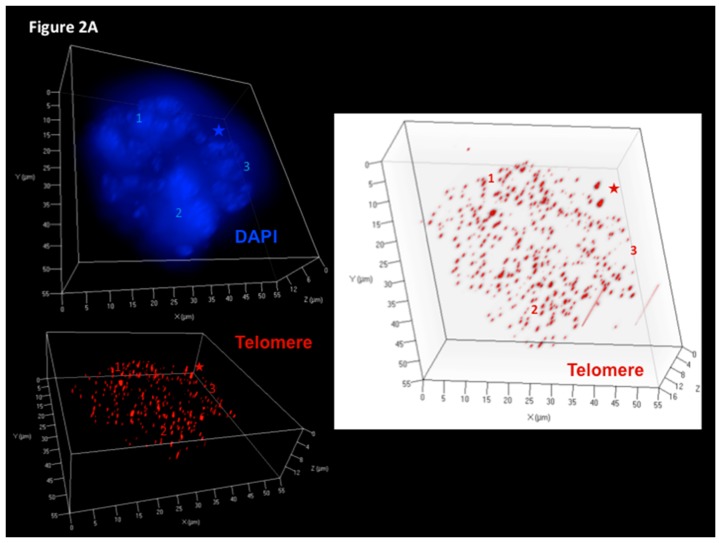

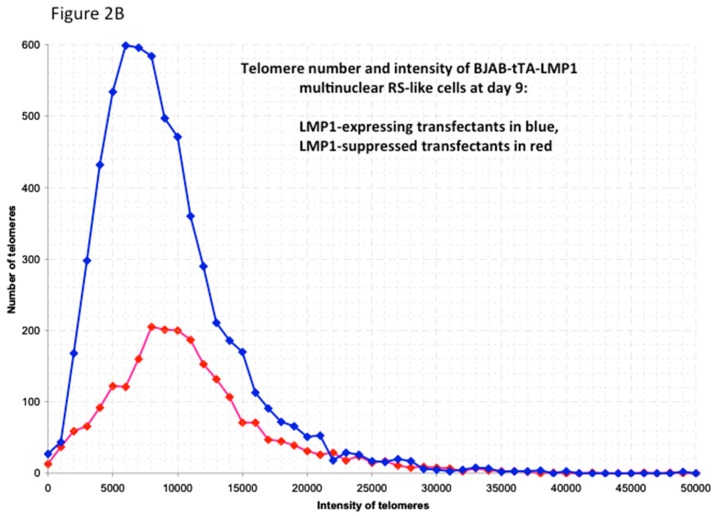
LMP1-induced telomere dynamics of multinucleated Reed–Sternberg (RS)-like cells. (**A**) 3D identification of disturbed nuclear telomere organization in a tri-nuclear LMP1-expressing Reed–Sternberg-like BJAB-tTA-LMP1 cell (upper left). Three-dimensional reconstruction of nuclear DNA (DAPI, blue) in surface mode reveals three nuclei (1–3). Three-dimensional telomere (red) reconstruction in surface mode (lower left) reveals abundant, irregularly distributed telomeres and two aggregates (asterix). Three-dimensional telomere identification in surface mode (right) against a white background (increases contrast and enhances visibility of short telomeres) identifies a total of 409 telomeres and confirms two large aggregates (asterix). (**B**). Telomere distribution according to size. Results are based on 3D analysis of 30 cells for each time point. Frequency (*y*-axis) and relative fluorescent intensity (i.e., size of telomeres (*x*-axis)) reveal individual telomere profiles at day 9. Telomeres with a relative fluorescent intensity (*x*-axis) ranging from 0 to 5000 arbitrary fluorescent units are classified as very short, with an intensity ranging from 5000 to 15,000 units classified as short, an intensity from 15,000 to 30,000 units classified as mid-sized, and an intensity >30,000 units classified as large [47]. LMP1 expression induces multinucleated RS-like cells with abundant very short and short telomeres already at day 9 when compared to LMP1-suppressed cells. Photomicrograph (**A**) and telomere plot (**B**) performed in parallel during the experiment shown in Figure 3 and Figure 4 of Lajoie et al. [46].

Figure 2A shows a 3D reconstruction of such a tri-nuclear LMP1+ RS-like cell with >400 telomere signals at culture day 7, and Figure 2B documents the 3D telomere dynamics of multinucleated LMP1+ RS-like cells in the Burkitt’s lymphoma cell line BJAB-tTA-LMP1 at culture day 9.

The dramatic changes in telomere dynamics are documented not only by a significant increase of cellular volume (<0.0001), number of telomeres per cell (<0.0001), and telomere aggregates (<0.0001), but also by a significant reduction of telomeres per 1000 μm^3^ of nuclear volume (0.007) [46]. Knowing that 3D-SIM imaging identifies large aggregates partially as clusters of (extremely) small telomeres [9], the kinetics are in favor of a substantial increase of very small telomeres (t-stumps). The most surprising findings are the LMP1-induced changes in expression levels of the shelterin RNAs and proteins, known to bind directly to the telomeres [46]. LMP1 expression rapidly reduces the TRF1, TRF2, and POT1 mRNA levels significantly (*p* < 0.05)—TRF1 and TRF2 from day 3 onwards, and POT1 from day 7 onwards. This suppression still persists at day 14. Moreover, this suppression is reversible, i.e., addition of tetracycline at day 3 or day 7 to the LMP1-expressing cultured cells completely restores the initial RNA levels measured at day one. Analogous findings are confirmed at the protein level by Western blotting [46]. The most prominent changes in LMP1 expression are identified in TRF2 RNA and protein kinetics: TRF2 protein is barely detectable in many RS-like multinucleated cells at day 14. Thus, we hypothesize that TRF2 reduction is tightly associated with multinuclearity. Proof that down-regulation of TRF2 is the key player in the formation of multinuclear RS-like cells is provided through blocking this LMP1-induced multinuclearity by LMP1 independent TRF2 expression [46]. 

When extending the analysis to the nuclear chromosome organization of BJAB-tTA-LMP1-expressing cells at day one and day 14 (supplementary material in [46]) using spectral karyotyping (SKY) [49] and comparing them to BJAB-tTA-LMP1-suppressed cells at day 14, significant differences are observed. In the LMP1 expressers, giant cells with complex chromosomal aberrations and up to 316 chromosomes, but also “ghost” cells with <20 chromosomes, are identified. On the contrary, BJAB-tTA-LMP1-suppressed cells show much less variation in chromosome number (between 44 and 58) and long BFB (breakage–fusion-bridge) “zebra” chromosomes [50] are significantly less frequent (5 in 15 cells compared to 21 in 18 cells for the LMP1+ multinucleated RS-like cells). In summary, in a germinal-center-derived B-cell setting, permanent LMP1 oncoprotein expression induces multinuclearity and is associated with the appearance of complex chromosomal abnormalities and formation of “zebra” chromosomes. Essential for this is the LMP1-induced down-regulation of TRF2—a key player at the chromosome ends [51].

## 5. Combined 3D Immuno TRF2/Telo-Q-FISH of Primary H and RS Cells

**Figure 3:**
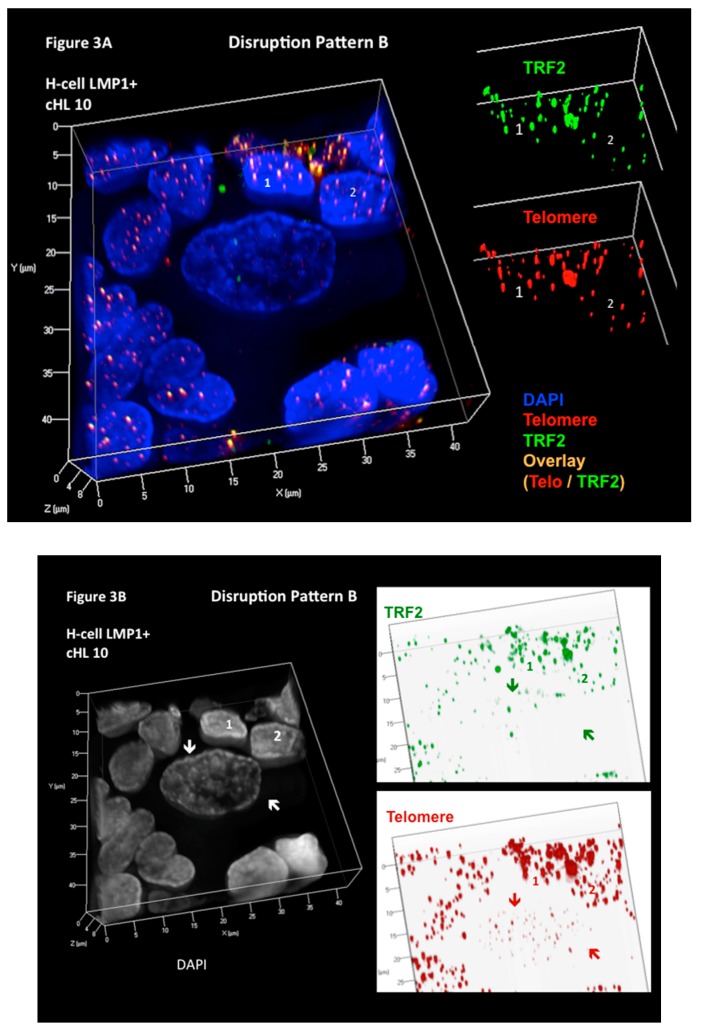
Three-dimensional Telomere de-protection in Epstein–Barr virus (EBV)-associated LMP1-expressing classical Hodgkin’s lymphoma (cHL) (disruption pattern B). Progressive attrition of telomeric repeat binding factor 2 (TRF2) spots occurs in LMP1-positive cHL during the transition from mononuclear Hodgkin (H) to RS cells. (**A**) Complete 3D reconstitution of large mononuclear H cell and lymphocyte corona (left) with nuclear DNA (blue), telomere (red), TRF2 (green), and telomere–TRF2 overlay (orange) signals is shown in transparency mode. The H cell shows several DNA-poor spaces and only few telomere and TRF2 signals, whereas the surrounding reactive lymphocytes (1,2) contain numerous small to midsized orange signals serving as internal control for tight 1:1 association of telomere/TRF2 signals. The TRF2 signal spots of lymphocytes 1 and 2 (upper right) and telomere signals (lower right) in surface mode show identical intensity and congruent localization in benign lymphoid cells. (**B**) On the left, the same complete 3D nuclear reconstitution (DAPI: white for better contrast) of large mononuclear H cell and lymphocyte corona (1,2) as in A. The two arrows serve as sentinel tags for better localization of the H cell on the right panel. Three-dimensional TRF2 (upper right) and telomere (lower right) identification in surface mode against a white background increases contrast and enhances visibility of short telomeres. The large H cell shows numerous small telomere signals without associated TRF2 signals and a partial dissociation of some TRF2 signals from the telomeres. Photomicrograph performed during case analysis as shown in Figure 4 of Knecht et al. [53].

To further test our hypothesis that the 3D interaction of telomeres and TRF2 is disrupted in H cells and directly associated with the formation of H and RS cells, and to further explore the aforementioned LMP1-mediated changes, we developed a combined quantitative 3D TRF2-telomere immune FISH technique (3D TRF2/Telo-Q-FISH) protocol [52]. We applied this technique to monolayers of primary H and RS cells, including surrounding reactive lymphocytes from diagnostic lymph node biopsy suspensions, allowing 3D analysis of the entire nuclear content [53], often not achieved using laser microdissection of H and RS cells, given that their nuclei are generally >10 μm in diameter and that this technique is performed on 5 μm sections. The results of 14 patient biopsies, four of them LMP1-positive, were just published [53]. Three additional biopsies (one LMP1-positive) have been analyzed (unpublished). The use of monolayers of whole cells has two major advantages: (i) the entire nuclear content of large, multinucleated RS is available for analysis; and (ii) the surrounding non-neoplastic (reactive) lymphocytes serve as an internal control. Our results show that the 3D steric interaction between telomeres and TRF2 is progressively disrupted from H to RS cells and that, surprisingly, two different (opposite) mechanisms are involved [53]. In the five EBV-associated, LMP1-expressing cases of cHL, marked loss of TRF2 signals physically linked to telomeres is observed. The signal-ratio telomere/TRF2 per case varies from 1 to 5.8 (mean 2.5) in the H cells and from 1.2 to 6 (mean 3.0) in the RS cells, resulting in progressive telomere de-protection during the transition from H to RS cells (Figure 3). This EBV- associated pattern (disruption pattern B) is also identified in four EBV-negative cHL biopsies.

**Figure 4:**
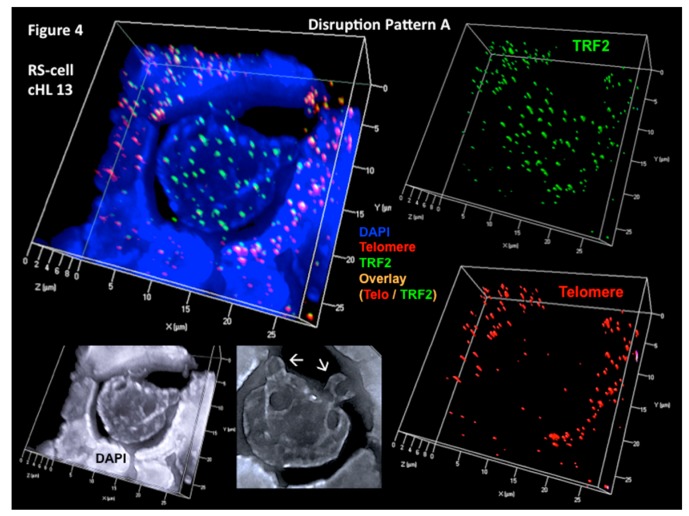
Substantial increase of unbound (free) TRF2 signals (disruption pattern A). Progressive shortening and loss of telomeres but increase of unbound (free) TRF2 spots occurs during the transition from H to RS cells as shown in this EBV-negative, aggressive cHL case. Complete 3D reconstitution of tri-nuclear RS cell and lymphocyte corona (upper left) with nuclear DNA (blue), telomere (red), TRF2 (green), and telomere–TRF2 overlay (orange) signals is shown in transparency mode. Mainly unbound (free) TRF2 signals are identified in the RS cell, whereas 1:1 telomere/TRF2 complexes are identified in the reactive lymphocytes. Lower left shows the same complete 3D nuclear reconstitution of the RS cell and lymphocyte corona (DAPI: white for better contrast). Arrows identify two satellite nuclei. Three-dimensional TRF2 reconstitution in surface mode (upper right) confirms numerous unbound (free) TRF2 signals in the RS cell when compared 3D telomeres (lower right). Again, the reactive lymphocytes show a tight 1:1 association of telomere/TRF2 signals. Photomicrograph performed during case analysis as shown in Figure 2 of Knecht et al. [53].

This direct 3D telomere–TRF2 interaction pattern corresponds to the one observed in our in vitro model for LMP1-induced H and RS cell formation in the setting of EBV-positive cHL [46]. On the contrary, in eight of the 12 EBV-negative cases of cHL, an attrition of telomere signals is associated with a massive increase of TRF2 signals no longer associated with telomeres. The signal-ratio telomere/TRF2 per case varies from 0.3 to 0.8 (mean 0.5) in the H cells and from 0.2 to 0.4 (mean 0.3) in the RS cells, resulting in an increasing number of free TRF2 signals during the transition from H to RS cells (Figure 4). Three of these cases had an aggressive clinical course, and in two of them DNA bridges between the nuclei of individual RS cells were observed (supplementary material in [46]). Analogous DNA bridges are documented in RS cells of the H cell line HDLM-2 [50,54]. 

## 6. Summary

Our findings in EBV-positive cHL confirm the in vitro observations that permanent LMP1 expression leads to downregulation of TRF2, telomere shortening, and multinuclearity. This mechanism is also observed in some EBV-negative cHL cases. Surprisingly, there is a second, opposite mechanism at work, associated with massive TRF2 upregulation and telomere loss. However, both molecularly disparate mechanisms converge at the level of 3D telomere–TRF2 interaction in the formation of RS cells, consistent with the hypothesis that cHL is a telomere-shelterin-related malignant lymphoma.

These above mentioned paradoxically opposite scenarios are supported by recent findings in molecular TRF2 research [51]. TRF2 deletion elicits an ataxia telangiectasia mutated (ATM)-mediated telomere damage response with γ-H2AX up-regulation, resulting in telomere fusions and consequently giant chromosomes in mouse fibroblasts [55] as well as in endoreplication and giant hepatocytes [56,57]. Thus, TRF2 expression is essential to avoid nonhomologous end-joining (NHEJ) recombination leading to giant chromosomes, hyperploidy, and endomitosis [55,58]. Indeed, γ-H2AX accumulation, giant chromosomes, hyperploidy, and endomitosis, are identified in RS cells of H-cell lines [50,59] and patient biopsies [53,59]. Thus, LMP1-induced downregulation of TRF2 (i.e., disruption pattern B) as found in EBV-associated cHL, is well supported by basic TRF2 research results.

On the other hand, TRF2 topologically stabilizes the 3′ single-stranded DNA overhang through DNA wrapping at t-loops [60], and is involved in the formation of t-loops at interstitial telomere repeat sequences which associate with lamin [61], primordial in the maintenance of 3D genome organization [62]. Most importantly, elevated levels of TRF2 induce telomeric anaphase bridges and rapid telomere deletions, emphasizing the importance of TRF2 for the interaction of 3D nuclear structure and function [63]. In this in vitro system, overexpression of TRF2 in HT1080 human fibrosarcoma cells rapidly leads to telomeric replication stalling, loss of telomeric sequences, and chromosome end-to-end fusions. Already after the first cell division after TRF2 over-expression, ultrafine telomeric anaphase bridges were observed, followed by significant telomere shortening after three to four cell divisions [63]. In analogy, DNA bridges between the nuclei of RS cells are identifiable in biopsies with EBV-negative cHL and significantly upregulated TRF2 signals (disruption pattern A) ([53], supplementary material) as well as in H cell lines [50,54]. Indeed, failing abscission without cytokinesis at the origin of binucleated RS cells, after division of mononuclear H cells, has recently been documented in time-lapse movies of the Hodgkin cell line KMH2 [64], thus confirming earlier observations that RS cells originate from single mononuclear H cells [65] and that RS cells do not represent cell fusions [66]. Furthermore, time-lapse movies identified apoptosis in H and RS cells [64], confirming that H and RS cells undergoing apoptosis are quite frequent (mean apoptotic index of 19%) in patient biopsies [67]. Whether or not the recently discovered telomere zinc finger-associated protein TZAP [68], which competitively binds (with TRF2 and TRF1) to telomeres, is associated with this rapid telomere trimming, is unknown. However, the near future will tell us whether TZAP plays an important role in the disruption pattern A.

In summary, the data presented in this review strongly support the notion of cHL as the first shelterin-telomere-related malignant lymphoma where LMP1 and TRF2 act as key players.
